# Social Justice Pedagogies in School Health and Physical Education—Building Relationships, Teaching for Social Cohesion and Addressing Social Inequities

**DOI:** 10.3390/ijerph17186904

**Published:** 2020-09-21

**Authors:** Göran Gerdin, Lena Larsson, Katarina Schenker, Susanne Linnér, Kjersti Mordal Moen, Knut Westlie, Wayne Smith, Rod Philpot

**Affiliations:** 1 Department of Sport Science, Linnaeus University, 351 95 Växjö, Sweden; lena.larsson@lnu.se (L.L.); katarina.schenker@lnu.se (K.S.); susanne.linner@lnu.se (S.L.); 2 Department of Public Health and Sport Sciences, Faculty of Social and Health Sciences, Inland Norway University of Applied Sciences, 2418 Elverum, Norway; kjersti.mordal.moen@inn.no (K.M.M.); knut.westlie@inn.no (K.W.); 3 School of Curriculum and Pedagogy, Faculty of Education and Social Work, University of Auckland, Auckland 1142, New Zealand; wayne.smith@auckland.ac.nz (W.S.); r.philpot@auckland.ac.nz (R.P.)

**Keywords:** physical education, health, equity, social justice, pedagogy

## Abstract

A focus on equity and social justice in school health and physical education (HPE) is pertinent in an era where there are growing concerns about the impact of neoliberal globalization and the precariousness of society. The aim of the present study was to identify school HPE teaching practices that promote social justice and more equitable health outcomes. Data were generated through 20 HPE lesson observations and post-lesson interviews with 13 HPE teachers across schools in Sweden, Norway, and New Zealand. The data were analysed following the principles of thematic analysis. In this paper, we present and discuss findings related to three overall themes: (i) relationships; (ii) teaching for social cohesion; (iii) and explicitly teaching about, and acting on, social inequities. Collectively, these themes represent examples of the enactment of social justice pedagogies in HPE practice. To conclude, we point out the difficulty of enacting social justice pedagogies and that social justice pedagogies may not always transform structures nor make a uniform difference to all students. However, on the basis of our findings, we are reaffirmed in our view that HPE teachers can make a difference when it comes to contributing to more socially just and equitable outcomes in HPE and beyond.

## 1. Introduction

School health and physical education (HPE) has the potential to support young people’s physical, cognitive, emotional, and social development [1,2] and contribute to them being/becoming physically active and healthy [3]. Indeed, education policy statements by the United Nations Educational, Scientific and Cultural Organization (UNESCO) [4] affirm our view that school HPE is linked to the education of “healthy” citizens and the development of physically active and healthy lifestyles that contribute to ongoing positive societal health outcomes. However, our understanding of what it means to be “physically active” and “healthy” is under constant (re)negotiation. In line with this special issue, recent decades have seen HPE curricula (in some countries) move away from the predominance of a scientific/physiological examination and explanation of physical activity, health, and the body to a more socio-cultural and socially-critical analysis and explanation. Socio-cultural and critical approaches to physical education, health, and the body are now foregrounded in HPE curricula in countries such as Sweden, Norway, New Zealand, and Australia [5,6] and require HPE teachers to integrate a socially critical perspective into their pedagogy [7,8].

Despite these messages that call for the adoption of a socially critical pedagogy in HPE, traditional biophysical notions of physical activity, bodies, and health are still being reproduced within this school subject [9]. Indeed, HPE continues to privilege and marginalise students on the basis of gender, sexuality, ethnicity, religion, and social class [10,11,12,13,14]. Although HPE has the potential to contribute to lifelong health and well-being, it can also have the opposite effect and be “unhealthy” for some students [15]. For some students, it can lead to experiences that cause them to view themselves in negative and unhealthy ways, which can marginalise them in terms of their social wellbeing. The rapidly changing student population that our HPE teachers are now faced with in their classrooms adds to the challenge of providing HPE curricula that strives for better health outcomes for everyone. A focus on equity, democracy, and social justice in HPE is pertinent in an era where there are growing concerns about the impact of neoliberal globalization and the precariousness of society [16]. While the relationships between neoliberalism, precarity, and health and wellbeing are complex and nuanced [16], the growth in rising inequality is well documented (see, e.g., [17]).

In many societies today, HPE teachers are being called upon to address significant social issues associated with low socio-economic lifestyle pressures such as family violence, mental illness, high levels of morbid obesity, and poorer academic outcomes from schooling, all of which are linked to poor health and wellbeing [18]. It is our view that these complex health issues require strategic *education* programs that take account of and aim to address the causes of health inequities, rather than deliver simplistic, interventionist physical activity programs that are designed to “make” children physically healthy. Over 20 years ago, prominent critical HPE scholar Richard Tinning [19] and others advocated for a critical approach to HPE that emphasized inclusion, equity, involvement, enjoyment, social justice, caring, cooperation, and movement. He argued that critical HPE should confront issues relating to gender equity, cater to diversity, and challenge unjust practices such as motor elitism. Subsequent advocacy for social justice pedagogies in HPE has been based on the premise that the subject is a site of educational practice where the reproduction of inequity, be it gender-, cultural-, or socially based, is either reinforced or challenged [20]. Regrettably, many HPE teachers are not sufficiently prepared to understand the issues and many do not have the necessary pedagogical skills to address and act on these social inequities [21,22,23].

This paper will present and discuss the findings of a study that was undertaken by a group of researchers from Sweden, Norway, and New Zealand. The study grew out of the researchers’ lived experiences as HPE teachers, teacher educators, and researchers in these three countries. As a collaborative group of researchers, we shared Tinning’s [24] belief that it is “how HPE teachers *think* and *feel* about education, social justice, physical activity, bodies and health” (p. 224, italics in original) that is of greatest importance in terms of promoting equity and social justice in HPE. An important focus of this study was, therefore, on how we can support our HPE teachers to embody socio-cultural understandings of physical activity, health, and the body, and enacting social justice pedagogies in their teaching of HPE.

Social justice pedagogies involve identifying inequalities and empowering individuals or groups to take social action to achieve change [25,26,27,28,29,30]. They are enacted when HPE teachers “seek to recognise and act on social inequities rather than further marginalise groups of students due to [their] e.g., gender, sexuality, ethnicity or socio-economic standing” [31] (p. 127). According to Wright [32], social justice pedagogies that foreground inclusion, democracy, social justice, and equity in HPE can assist students to examine and challenge the inequities in the status quo, the dominant constructions of reality, and the power relationships that re/produce inequities in ways that can lead to advocacy and community action. Social justice pedagogies can be transformative when they help students develop an awareness of their own perspectives, and come to see the world in different ways [33]. The process of transformational learning relies on pedagogies where learners are given the confidence to reflect on their values, social norms, and assumptions [34].

Research advocating for social justice pedagogies in school HPE and teacher education has been on the agenda for several decades and continues to thrive in many countries including the USA [35], Canada [36], Australia [24], New Zealand [37], and Sweden [22]. Unfortunately, this advocacy for social justice in HPE has not been matched with examples of how HPE teachers can actually teach for social justice, that is, what teachers can do in their classrooms, and for whom social justice is sought [21]. In one of the few HPE classroom accounts, in which they studied teachers’ practices relating to the experiences of girls in HPE lessons, Oliver and Kirk [38] drew on an activist research approach to identify four critical elements that need to be present in the teacher’s pedagogy in order to assist girls to identify, name, and negotiate barriers to their engagement in HPE and embrace a physically active lifestyle. These were a student-centered pedagogy, opportunities to study issues of embodiment, inquiry-based learning, and sustained listening and responding. In another example of a study of (socially) critical pedagogy in practice, Fitzpatrick [39] shadowed a HPE teacher, Dan, as he taught HPE at a high school in New Zealand. Fitzpatrick [39] described Dan as being “passionate about critical pedagogy” (p. 80). She then described the key tenets of Dan’s critical approach and success, which were “building the environment; deconstructing power; playfulness; studying critical topic; and embodying criticality” (pp. 193–206). Similarly, Lynch and Curtner-Smith [40] reported on the social justice pedagogies of a single HPE teacher in the USA, which included a focus on nurturing trusting relationships, using student voice, studying critical topics, and using alternative assessments. In an Australian context, a recent case study by Alfrey and O’Connor [41] reported on how a large HPE department in Australia worked alongside a group of researchers to transform their secondary HPE to align with the critical intentions of the Australian HPE curriculum. Whilst the authors acknowledged the challenge of transforming practice, they concluded that the transformation in the teachers’ practices was facilitated by providing them with the time and space to engage in repeated action and reflection. They also reported that this “continues to be transformative of their identities and philosophies as HPE teachers” [41] (p. 12).

Notwithstanding the importance of these research findings, there is still a paucity of studies documenting and providing empirical evidence of teachers’ enactment of social justice pedagogies in school HPE across varying contexts. The aim of this study, therefore, was to address this knowledge gap and contribute to our understanding of how teachers in HPE can teach for social justice by examining their teaching practices. Hence, the overall research question guiding the study was: *How do HPE teachers’ practices address social justice?*

## 2. Materials and Methods

### 2.1. Participants

The study participants were 13 teachers from four schools in Sweden, three schools in Norway, and four schools in New Zealand. The teachers were selected through purposive sampling [42]. They were required to be qualified and fully registered teachers with a minimum of three years full-time teaching experience. The teachers were familiar to the research team and selected as examples of HPE teachers known to foreground social justice in their teaching practice. The seven male and six female teachers ranged in age from 25–55 with between 3 and 25 years teaching experience. The observations were restricted to compulsory HPE classes with 13−15-year-old students in co-educational schools. Of the 11 schools, 10 were public schools. One school was a “charter” school in a low socio-economic area in a small Swedish town. A multicultural school demographic was a common feature of the participant schools in all three countries. Table 1 provides an overview of the research participants and school context. In order to protect the anonymity of the participants, all teacher names referred to in this paper are pseudonyms.

### 2.2. Data Collection

The data reported on in this paper were generated through 20 HPE lesson observations and interviews with 13 HPE teachers across schools in New Zealand, Sweden, and Norway.

The data collection was based on the principles of critical incident technique (CIT) methodology [43] and stimulated recall interviews [44]. CIT was developed to capture not only the actions, but also the thought processes and the perspectives of teachers in relation to critical incidents. The logic of CIT rests on the assumption that not all actions (“incidents”) are equally important, with some incidents being exponentially more important than others. Therefore, the strength of CIT is its high degree of focus on “*things that matter*” in a particular activity [45]. In this study, we employed CIT to explore the thought processes and actions of HPE teachers with a narrow focus on teaching for equity and social justice (for a full discussion of the methodology used in this study, see [46]).

All observations were completed by at least two researchers (including a minimum of one host and one visiting researcher) using an observation template (see Table 2) informed by literature on critical pedagogy, transformative pedagogy, and teaching for social justice. Data recorded included a description of the context, the teacher, and the class and descriptions of “captured incidents” including the actions of the teacher; teaching artifacts; the response of the students; and, in the final column, initial coding of the type of social justice issue that may have been addressed (based on the previously mentioned literature). “Captured incidents” were incidents worthy of further exploration as a possible social justice pedagogy. Captured incidents were later confirmed as “critical incidents” on the basis of clarification from the teachers as to the purpose of their actions.

Our premise for adding the concept of “captured incidents” and interviews to our methods was that observations alone are not sufficient to understand the thinking behind teacher actions nor the intended consequences. To gain a deeper understanding of the teachers thinking, we questioned the teacher about what we had observed through subsequent stimulated recall interviews. The interviews lasted 40−70 min and took place immediately after, or almost immediately after, the observed lessons. The interview guide involved a combination of open questions designed to enable the teacher to elaborate on social justice pedagogies, as well as specific questions designed to afford the teacher an opportunity to explain the thinking behind the identified captured incidents.

### 2.3. Data Analysis

The data, both observation notes and interviews, were analysed following the principles of thematic analysis [47,48], informed by our existing knowledge and positioned within the paradigm of critical qualitative research for social justice [49]. Data were at first analysed separately in each of the three national contexts (Sweden, Norway, and New Zealand). The first two phases of analysis involved familiarisation with the data and individual open coding [48] of the observation notes and transcripts by all members of the research team. Once individual researchers had read and thought about the data, through a process of sifting and sorting [50], initial themes were identified and named. Researcher pairs from each country then met to compare, cross-check, and reduce initial codes and themes into common/shared codes and themes. The third level of analysis was a group analysis by all members of the research team. Initial themes from each country were shared electronically and discussed as a group through an online video conference meeting. All of the themes were then mapped against each other by the lead author, reframed on the basis of the research question as three themes, and distributed to all members of the research team. A final group meta-analysis was conducted to ensure that the themes satisfactorily captured the perspectives and interpretations of all the participating researchers. These three themes were (i) “relationships”, (ii) “teaching for social cohesion”, and (iii) “explicitly teaching about and acting on social inequities”.

### 2.4. Ethical Approval

The research project was granted ethical approval in Sweden by the Regional Ethical Approval Committee in Linköping (DNR 2017/201-31); in Norway by the Norwegian Centre for Research Data (REF 53653); and in New Zealand by the University of Auckland, Human Participants Ethics Committee (REF 019009).

## 3. Results

In this section of the paper, we present and discuss the findings relating to the three themes: (i) relationships; (ii) teaching for social cohesion; (iii) and explicit teaching about, and acting on, social inequities. Collectively, these themes represent the enactment of the social justice pedagogies that we identified in our project. The themes are purposely presented in this order because we believe that in most classrooms building relationships precedes or occurs concurrently with teaching for social cohesion, providing foundations that ultimately enable explicit teaching about and acting on social inequities in HPE practice. On the other hand, we also believe that they are all interrelated and, in some situations, taking action on inequity is the catalyst for building a trusting relationship with students. As such, we propose that HPE teachers who are interested in and committed to the social justice agenda should consider taking each of these three elements into account as a part of their social justice pedagogy.

### 3.1. Relationships

In this section, we deal with the first theme—“relationships”—as our data clearly show that teaching practices aimed at building relationships are fundamental to pedagogies for social justice in HPE:

All teacher participants recognised the importance of building relationships. Many of them talked about the importance of getting to know their student(s). One teacher, Louise (Sweden (SWE)), reflected this common view when she said, “I think if I know them and talk to them, we show respect to each other”.

Despite the different cultural and societal contexts of each country, the teachers emphasised the need to know the everyday lived experiences of their students. For example, Dillon (New Zealand (NZ)) talked about the value of both living within the school community and understanding the implications of socioeconomic differences:

*“Being in the HPE [section] for the last four years, and listening and talking to parents and understanding and living here, I know for some of them [the families] financially it is not possible [to buy a HPE uniform], and so I am not going to punish them because they can’t afford to buy … a uniform.”*

(Dillon, NZ)

This quote illustrates how Dillon’s knowledge about the students’ local community guides his practice as a HPE teacher. Dillon’s acknowledgement of the socioeconomic conditions that many of the students at this school live under has a greater influence on his practice than strictly following the school’s HPE uniform regulations.

Knowing the students on a personal level was also important for most of the teachers in this study. Phrases such as “focusing on the individual”, “seeing each student”, “I have to know them”, and “closeness to the students” were common features in our data. The teachers also reported using different strategies in order to get to know their students. Several of the teachers, for instance, talked about the importance of knowing all their students’ names. Candice told us how she “played a really boring name game at the start of the year…[but] it was actually perfect because now I know all of their names… it makes a big difference” (Candice, NZ). She also suggested that knowing the wider families was equally important. Candice stated that, “I know a lot of their older brothers and sisters, which has actually helped as well because they can relate to me”.

During our observations and interviews, we identified how the teachers aimed to build relationships through employing caring teaching strategies. Our findings reveal how these strategies had the intention of building relationships as a basis for socially just teaching. Caring teaching strategies in this regard were incidents where we recognised that the teachers performed actions that were caring when interacting with their students. For example, we made the following observation when observing Emma in Sweden “[She provides] additional support services with the teacher showing a high level of care and concern to ensure positive integration as much as possible” (Observation notes, Emma, SWE). We also identified that some teachers combined the caring part and the teaching part of the job. For example, when we observed Kendall (NZ), we found that besides being organised and well prepared, “she also stood out as a ‘mother figure’” (Observations notes, Kendall, NZ). In the interview, she elaborated on this:

*“I looked at these kids and went wow…some of these students don’t have structured parents … I felt it was something that was almost like combining a little bit of parenting with a little bit of teaching.”*

(Kendall, NZ)

Another important aspect of building relationships was the consistency through which all participant teachers engaged in positive ways with their students. In one particular case, we observed how the teacher (Gary) started and ended the lessons by praising the students in different ways. When we asked Gary about this he said:

*“I guess that is something, that me looking back as a student and as a person in general, that I think when someone is positive to you, you have an inclination to be quite positive yourself and you feel as if you have done something well and it sets the tone, I think, for the lesson and for yourself as a person. When you hear those things to just get off on the right foot, I think, and it is something not just teaching wise as an interaction as a social thing. I think it is really important to start with something positive.”*

(Gary, NZ)

At one of the Swedish schools, we also observed how the teacher (Charlie, SWE), used a lot of physical touching. When asked about this, Charlie said:

*“At this school it is common with physical contact in the form of hugs and touching when you are talking to the students…Many of the teachers at this school use touching as a pedagogical tool. It’s very natural for me and my colleagues. We feel that the students need this form of contact. It is a form of comfort and confidence building but not everyone likes it so we only do it when we know it is ok… It is a way of communicating and is a common approach in a school with many different ethnicities and they need this. It is another way of communicating when you don’t know the language…I think you can use it to calm some students by touching them. They get a little calm then.”*

(Charlie, SWE)

Another aspect of practice that highlights the importance of relationship building as a social justice pedagogy was how the teachers used their knowledge of the different abilities and sporting interests of the students in their class to design inclusive learning activities. The following quote from Per (Norway (NO)) highlights how he used team building activities to build group understanding and tolerance:

*“…We have worked on, call it team building activities. It is the second year I have them [this class] … we have worked a lot with activities to avoid … unintended conflicts between groups in the class.”*

(Per, NO)

In order to avoid conflicts between groups of students, Per claimed the teacher needs to know the group members and consider the relationships between students when planning and delivering the lessons. Charlie (SWE) similarly explained how she purposely used her knowledge about the students’ group dynamics when she divided the class into different groups:

*“I always mix students when I divide them … I have noticed that they behave in different ways when they are with their best mates and when they are with someone else.”*

(Charlie, SWE)

Our overall finding related to the theme of relationships is that the teachers in this study stressed that, “*it is [their] job as a teacher to build good relationships with the students*. *This is not a student job”* (Per, NO). Additionally, many of the teachers highlighted that good relationships are only established over time and through personal investment in the relationship. As Dillon (NZ) summarises, “it takes time to build a relationship”. For these teachers, knowing the group and acting on this knowledge is essential to building relationships between student and teacher, and critical for creating a socially cohesive classroom.

### 3.2. Teaching for Social Cohesion

This section focuses on the second theme, “teaching for social cohesion” and highlights the teachers’ related pedagogy as well as the significance the teachers gave to addressing social cohesion in their lessons.

When teaching for social cohesion, many of the participant teachers demonstrated a focus on inclusiveness and the importance of establishing an inclusive class environment, with several stressing the need for them to ensure that they included all students to make them feel that they belonged. Inclusiveness was most often generated by the teachers’ own positive and supportive attitude towards their students, or in their planning to provide meaningful enjoyable experiences. For example, in her interview, Kari stated that “I want everyone to be successful. When everyone is successful, they feel part of the group. The students’ experiences matter most with the student’s self-achievement being most important not performance outcomes” (Kari, NO). When teaching, Kari presented a positive disposition, which she wanted to be infectious. As she said, “enthusiasm gives enthusiasm back again” (Kari, NO).

Another key feature of teaching for social cohesion that the participant teachers recognized as being important was the use of culturally inclusive pedagogies. For example, in New Zealand, two of the teachers included the indigenous Māori language and Māori values in their lessons. For example, Kendall “used te reo Māori (Māori language) on a wall chart and on the whiteboard. ‘He mahi tahi tatou mo te oranga o te katoa—which means ‘we should all work together for the wellbeing of everyone’” (Observation notes, Kendall, NZ). Tane, also used te reo Māori during his lesson, with directions such as “Haere mai (come here), taonga (the treasured ball in the middle)” (Observation notes, Tane, NZ). In Sweden, Kane, who had been teaching classes of new immigrant children for two years, also recognised the need for culturally inclusive pedagogy. He recognised that he had to change his practices to make his classes inclusive and enable student success. Kane stated:

*“when I started working [at this school] I started with full immigrant children and I had to adapt my way of teaching to that with body language and always showing or using other students to show them how to do it.”*

(Kane, SWE)

These teachers showed that they were aware that cultural sensitivity and critical consciousness are necessary starting points for building social cohesion across multiple groups and for addressing deeper issues of equity and social justice. They were very aware that their inclusive pedagogy had the potential to increase trust, acceptance of diversity, and full participation, but perhaps more importantly, to break down social barriers between different groups.

Indeed, many teachers showed that they wanted to develop cooperative student-to-student interactions in their lessons. Most teachers purposefully and carefully mixed students and used multiple-paired activities to maximise the range of student to student interactions. For example, Kari used “Cross-group mixing to encourage social integration… and different combinations to encourage intra-group diversity and to help the students understand the nature of diversity within their own class” (Observation notes, Kari, NO). In many cases, the activities that the students engaged in were just a vehicle for learning another’s social values and fostering social cohesion:

*“Social cohesion and building social responsibility overrode all other content objectives to the point that the nature of the activity (Turbo touch) was just a known and enjoyable medium for teaching the more important ‘bigger matters’ of establishing strong social cohesive values. The enjoyable game enabled social collaboration and a medium for developing socially acceptable practices and in this case self-management within this collective.”*

(Observation notes, Candice, NZ)

The nature of the pedagogies used for teaching for social cohesion also frequently involved physical contact between the students, with students holding hands during game activities or physically supporting one another during problem-solving activities. Kari used “a tag game [that] required students to hold hands and work with a partner BUT rather than stay with the same partner, the pairs [had to] split every time a tag [was] made” (Observation notes, Kari, NO). The lessons also involved deep discussions among groups of students, as they discussed and negotiated who would do what, what was fair, and what was not, etc. For example, Charlie, “asked the students to go to their places on the court in their teams and discuss how they [could] work together (collaborate) in the game they [were] playing” (Observation notes, Charlie, SWE). Indeed, team building, heterogeneous teams, and cooperative games were a cross-nation feature of most HPE classes we observed. In Norway, we observed Kari:

*“working on team building, that is, how to work together as a team. She uses cross-group mixing to encourage social integration through working together for the common good of all in the team. Uses different combinations to encourage intragroup diversity and to help the students understand the nature of diversity within their own class.”*

(Observation notes, Kari, NO)

In her post-lesson interview, Kari said:

*“The aim of the session was to do activities where they must work together and do something together… with a view to building a safe and happy environment…Also, I like to add the [rule] where they have to change partners after each, and be with more, in that I want to build this social community and the relationships between pupils…then they must even more talk together and make a plan and perhaps discuss a little to solve the task together.”*

(Kari, NO)

When teaching for social cohesion, many of the teachers felt that they had a role to play in helping their students become more responsible for their actions. In many of the classes we observed, the teachers focused on developing personal and social responsibility. In this endeavour, many of the participant teachers provided the opportunity for the students to work independently in supervised but trusting environments. They encouraged student choice with the expectation that the students would accept this and behave responsibly. In one critical incident the teacher, Candice, selected mixed teams, then:

*“Sent the students off to organise their own small-sided games of a game they knew well. She expected the students to take responsibility for their own actions and play fairly and to support or caution others when necessary. She asked them to organise their own captains, set up the equipment on the field, begin the game and referee themselves. At the end of the lesson she gathered the class in and asked ‘How did you behave? How can you work on your personal and social responsibility outside of the PE lesson, in life?’”*

(Observation notes, Candice, NZ)

Kari also used team and cooperative games to teach personal and social development. At the beginning of her lesson:

*“she and the students discuss the meaning of fair play so that they all have a common understanding… Students share in discussion to decide the nature of fair play so it is an opportunity for them to participate in a democratic process for the good of all. The teacher supports the lesson objectives with related questions about fair play.”*

(Observation notes, Kari, NO)

In another critical incident observed at a school in Sweden, Emma asked students to work independently in groups to create fitness activities with differentiated levels of difficulty:

*“She had trust in them. She asked each student to create an activity for the others to follow. After leaving them to come up with the activities she then joined in and commented on the chosen exercises.”*

(Observation notes, Emma, SWE)

In her post-lesson interview, she said that she knew “they would be able to complete the task of designing the activities for the lesson” and “they are used to this expectation of independent learning and taking responsibility for their own learning” (Emma, SWE). Emma suggested that she had built strong relationships with the students over a number of years and they had developed confidence in working independently in small heterogeneous groups.

In summary, this section highlights how the participant teachers’ teaching for social cohesion involved a focus on inclusiveness, the inclusion of cultural inclusive pedagogy; using team building, heterogenous teams, and cooperative games; and developing personal and social responsibility. In effect, these teaching strategies can be seen as examples of the teachers’ enactment of pedagogies for social justice.

### 3.3. Explicitly Teaching about and Acting on Social Inequities

This section reports on the third and final theme, “explicitly teaching about and acting on social inequities”:

Many of the participant teachers in our study recognized that teaching for social justice and acting on social inequities requires an uneven distribution of resources. Candice, for instance, stated that equity is “not necessarily treating everyone the same but you still having that same expectation …”. She explained further:

*“I guess things are not always black and white there is grey in that it is not one size fits all so like maybe they are not as good as that person but they are putting the effort in or they are improving like that is important but I think that it can get lost sometimes as teachers because it is easier if everybody is doing the same thing but I think if anything in terms of social justice then we need to be a bit more flexible and allow for difference”*.

(Candice, NZ)

In Norway, Kari modified the rules of a game for a student who was not in HPE clothes and was not participating due to injury. When reporting on this lesson one researcher noted, “I overheard [the] teacher asking one boy who had surgery on his foot if he could attend if he were allowed to walk (not run) instead of sitting on the side. He answered yes. Then she made up a rule that he could walk, and instead of one hit, the students had to hit him twice (he had “two lives” in the game) before he was out.” (Observation notes, Kari, NOR).

In all three countries, the teachers purposefully made exceptions to rules around appropriate HPE uniform, exceptions that at times were likely to contradict broader school rules. In schools in lower socio-economic areas, where we collected data, the willingness of teachers to make exceptions was most often based on the belief that the students should not be punished for socio-economic or health reasons. As mentioned previously, Dillon (NZ) stated that, “I am not going to punish them because they can’t afford to buy a uniform”. Charlie (SWE), who similarly taught in a school in a low socio-economic area, indicated that these exemptions resulted in greater inclusion:

*“…if they have forgotten their training clothes they are allowed to participate. We have also bought some clothes that the students can borrow because there are some students who don’t have any clothes. It is very seldom some of the students do not participate.”*

(Charlie, SWE)

Promoting marginalized groups was another key feature of how the participant teachers purposefully addressed social inequities in their teaching practice. One example of this was how HPE teachers in New Zealand incorporated te reo Māori (Māori language) in their lessons. In the interview after one these lessons, Kendall (NZ) acknowledged that her actions had a strong social justice agenda:

*“It is about doing it [incorporating tikanga or Māori culture] in a way that is authentic rather than, I could have written the date up in Māori but actually that is a tokenism kind of way of doing it. So, what I am trying to do is introduce that sort of culture into the class in a way that is more authentic...I think as educators, we have a responsibility to try and bring that [Māori culture] back in.”*

(Kendall, NZ)

In Sweden, where swimming ability is assessed to a specific standard in the HPE curriculum, many of the teachers in the study described how they actively try to support the large groups of newly arrived refugee students who typically have not come from swimming backgrounds. Kane explained how the students who were new to Sweden received extra support:

*“At this school we have 40 or 50 girls who only swim with other girls…So the way around it, we found, was one time a week, one morning before school or around when school starts, one of the staff here at school takes them there [to the community pool] and helps them with the technique … The results are very, very good.”*

(Kane, SWE)

In New Zealand, promoting of marginalized groups also involved explicitly teaching about social inequities in relation to physical activity, sport, and the body. In one of the lessons, we observed the teacher (Tane) who, after having watched the movie “Bend it like Beckham” discussed with his students how gender and different ethnicities and cultural identities may enable or limit participation in physical activity and sport. In his interview, Tane said,

*“The learning behind that lesson was looking at ethnic and gendered identities within sport and how that might influence you to take part in different types of sports as opposed to others and looking at what influenced those identity in the first place…in the second class I did it through actually playing different sports and then trying to ask why do they enjoy these sports and then look at the experiences as to what created or helped shape their sporting identities and why that might be how they enjoy certain sports over other sports.”*

(Tane, NZ)

In this statement, Tane suggests that raising awareness of existing social inequities in physical activity, health, and the body was seen as an important part of learning in HPE. However, it is less clear if Tane takes further steps address the inequities after raising this awareness or how this might influence his teaching practice.

Another way the teachers acted on social inequities in their teaching was through their own critical reflection on HPE practice that moved beyond merely technical aspects of teaching. Ola (NOR), an early career HPE teacher, critically reflected on how he tries to reduce the power relationships in his class when showing his awareness of his own positionality and how this affects social justice pedagogies in his classroom:

*“A teacher is in a position of power…in terms of communication and relationship building, I cannot stand there and work from the top down the whole time. I have to meet the students and... just by bending me down I can change the balance of power a little.”*

(Ola, NOR)

In the following statement, Dillon (NZ) critically reflects on how the actions he takes in class are based on his own life history, which included his own unpleasant memories of schooling that he does not want to reproduce. He stated:

*“I went to a boarding school when I was in Africa so I got caned quite a few times and the teachers would always yell and I hated school, I mean I really hated school. And then when I moved to NZ, I had a couple of teachers that were just calm, they still have the behavioral issues, but they wouldn’t ever be in your face yelling. I want to be a teacher that doesn’t do that. I just want to be calm I just want them to understand where I am coming from. I don’t think I have ever yelled in eight years and kids understand that. They respect it.”*

(Dillon, NZ)

In Sweden, Emma similarly recounted how her own experiences during her HPE teacher training had shaped her teaching practice to first and foremost focus on making the students feel safe. In her interview, Emma told us how one of the other students had been treated badly and left by himself by the teachers when out cross-country skiing:

*“I lived with him in the same cottage…he could not do anything like he fell everywhere and he could not handle the situation at all…he was more and more hurt because he fell more and more and he was up there on that mountain and he was just in like jeans or something or like a leather coat, you know…and then our teacher disappeared to the good group…so some of us students had to like help him out…so that feeling was not nice and I didn’t like how they treated him and I did not like how the teacher did not step up like took the responsibility for his safety…so now when I am teaching my first priority is always to make them [the students] feel safe.”*

(Emma, SWE)

These are examples of teacher critical reflection that involve reflection on issues of power, care, and safety that remain at the heart of social justice pedagogies in HPE. Ola and Dillon are conscious of power relations in relationships between students and teachers. Emma is conscious of her own life history and she reflects on how these previous experiences have influenced how she relates to her students.

## 4. Discussion

The aim of the present study was to explore HPE teachers’ practices that address social justice in three different countries. The findings presented in this paper have shown how social justice pedagogies in HPE can be enacted through *building relationships*, *teaching for social cohesion*, and *addressing social inequities*.

Firstly, *building relationships* can be seen as an initial critical element of socially just HPE teaching. Findings from this study demonstrate how building positive relationships is a process that requires the teacher to develop knowledge about their students. Many of the teachers in our study focused on developing knowledge about the students at a personal level, for example, knowing their names as well as the names of their siblings, and more generally showing an interest in the students’ personal biographies because they recognised that this knowledge helps deepen their relationship with their students [51]. The teachers also drew on their knowledge about the non-homogenous nature of the students and integrated this knowledge into their teaching practices. Other relationship-building strategies used by the teachers in this study, such as saying “hi”, sitting down on their level when speaking, forming and being part of the circle, using physical touch, and generally being positive and encouraging, can be seen to be pedagogical tools to show empathy and care for students [52,53]. If HPE teachers employ these teaching strategies for building relationships, we believe that more inclusivity and teaching for social justice occurs [54]. However, again, it is important to add that although building relationships can be seen as a “foundation” for social justice pedagogy [40], social justice pedagogy requires a further step to engage in “pedagogies of discomfort” [55] (p. 517), that is, to examine and disrupt taken-for-granted practices that create social inequities.

Secondly, the findings of this study exemplify the HPE teachers’ focus on *teaching for social cohesion*. Teaching for social cohesion can also been seen as both a precursor and necessary first step towards teaching for social justice. When pupils limit their interactions to peers with the same cultural backgrounds in school, there is less opportunity to educate for social cohesion [56]. Learning tasks in HPE that require collaboration in mixed groups can therefore be seen to be important for the students’ understanding of each other [57]. The participant teachers in this study recognised that all students need to build relationships and trust, as well as learn to value others, and they therefore purposely and carefully structure classroom learning experiences in a range of contexts including minor games, outdoor pursuits, and modified sports to build social cohesion. Similar to previous studies that have examined how HPE teachers strive for social cohesion (see, e.g., [57,58,59]), the findings in this paper highlight how teachers provide opportunities to work in heterogeneous groups and to problem solve in these mixed group settings and cooperative games, and perhaps, most importantly, how they developed their practices in culturally responsive ways. In this sense, HPE provides an ideal interactive learning context for the teaching of social cohesion and through extension social justice by providing active learning experiences where students can learn with and about other students with whom they may not normally associate with in their daily lives. This study has served to reinforce our belief that the very nature of compulsory school HPE highlights its mandate, responsibility, and role in educating for social cohesion [60]

However, it must be added that social cohesion is not about learning to be the same or hold the same values as all others in the class, but about learning about differences and learning to live with, get on with, and tolerate and accept both similarities and differences between groups and individuals. It is a sense of belonging that is created when individuals in a group simultaneously embrace diversity as a catalyst for growth and learning while valuing aspects of common identity that bind them together. Educating for social cohesion is about building socially acceptable coherences between individuals or groups who may be very different and hold different views, values, and practices. As we have seen in this study, good teachers are the catalyst for social cohesion within a class, but the greater benefits occur when the students transfer these values into their everyday lives in their own communities and work places.

In this sense, we want to stress that teaching for social cohesion as a quest for, or precursor to, social justice must move HPE teachers’ practices and students’ thinking beyond mere social integration. It also needs to address the social conditions that simultaneously privilege and marginalise the values and beliefs of certain groups in society [61]. For this reason, whilst the examples of teaching for social cohesion identified in this study are important, HPE teachers should remain mindful of the requisite conditions and processes in which social cohesion may develop. These include Allport’s [62] and, more recently, McKay’s [63] calls for learning activities that promote equal status, cooperation, and agreement on social norms and Pettigrew’s [61] advocacy for practices that provide sufficient interaction to enable “ingroup” students to learn about and develop affective ties with the “outgroup” students in order to appraise their own beliefs and change their behaviours.

Thirdly, the teachers’ examples of *addressing social inequities* provided in this paper demonstrate a move beyond teaching *about* social justice issues for the purpose of increasing student consciousness of social justice issues [64]. As intended, the findings of this project are examples of the actions of teachers who aim to teach *for* social justice. In addressing social inequities, these teachers are acknowledging the challenges faced by the students in their classroom contexts and, in response, they aim to create classroom climates that embrace multiple perspectives and provide equitable opportunities for learning. In contrast to the suggestion that “[H]PE is an exclusionary and marginalized space for many students” [65] (p. 1129) and that “[H]PE is too competitive” [66] (p. 171) and focused primarily on health and wellness [67], most lessons we observed involved playful activities with a limited focus on performance, positive interaction between students, and significant student input through modified activities and rules, as well as challenge by choice approaches in activities such as canoe rescues. These lesson environments do not happen by chance, but rather they are organized in ways that create more inclusive, fair, and equitable embodied learning experiences for young people [16]. Many of the examples we have presented in this paper are of teachers who recognize inequities and choose to challenge them. Notwithstanding claims that HPE can still make friends and enemies [68], our findings reaffirm our belief that HPE can be a learning space that is inclusive and can educate for social justice outcomes.

Although our findings provide examples of teaching practices that move beyond advocacy and address social inequities, we recognize that the presentation of practices as themes and subthemes can serve to disguise both the importance of knowing and understanding context and the tensions inherent in enacting social justice pedagogies across different socio-cultural and political contexts. The explicit examples of social justice pedagogies that are described in this paper are performed in nuanced contextual/relevant ways that focus on addressing social justice issues most relevant to the particular context [69]. In Sweden, the teacher actions focused on providing equitable opportunities and outcomes for new migrant students and in Norway, strong social democratic principles appeared to predicate the teachers’ inclusive practices. In New Zealand, a strong focus on indigenous Māori culture was observed. The examples of teaching practices we have observed and explored in interviews were focused on social justice issues that are found in that particular school context, as shaped by the surrounding society and curriculum. These teachers have identified and addressed social inequities in their particular class/school by promoting/enhancing the experiences and outcomes of marginalized groups, but this goes beyond the mantra of equality of opportunity to a focus on achieving greater equity in HPE [70].

Finally, while the findings presented in this paper are mainly examples of explicit social justice pedagogies aimed at building relationships, teaching for social cohesion, and addressing social inequities, we suggest that teaching for social justice requires teachers to both teach *for* and *about* social justice. Teacher actions that act on social inequities in school without explicitly talking about social inequities run the risk of leaving students to be ill-prepared to be their own agents for change and act on social inequities beyond HPE. Rather than simply teaching for social justice, teaching about social justice requires teaching that raises student’s critical consciousness so that the students can reflect on and develop their agency/disposition to engage more knowingly in society. We should remain conscious that teaching for social justice by taking action on inequity without educating about deeper societal issues that create inequity may not cultivate the consciousness required to empower advocacy and community action [32]. We therefore call for the further development of social justice pedagogies in HPE that involve the problematization of knowledge construction and how the dominant ways of thinking about physical activity, health, the body, and self have come to be, and where students are challenged to change the structures that create social inequities [24,71].

One limitation of this study is the small sample size coupled with the fact that we selected the participant teachers through purposive rather than, for instance, random sampling [42]. The teachers were familiar to the research team and were chosen for the study on the basis of our experiences working with them in HPE teacher education programs as examples of HPE teachers more likely to be inclusive, reflective, and willing to challenge social norms and inequities in schools. As such, we cannot make any generalization or claims about the practices of all HPE teachers. However, we still believe that our study provides some important insights into how social justice pedagogies can be enacted in HPE. Another limitation is that our study did not include student’s experiences of these social justice pedagogies being enacted by the teachers. Future studies could therefore both include a larger and more varied sample of teachers as well as an examination of the students’ experiences of social justice pedagogies in HPE.

## 5. Conclusions

In conclusion, this paper demonstrates the enactment of social justice pedagogies in school HPE through building relationships, teaching for social cohesion, and explicitly teaching about and addressing social inequities. We recognize that the examples of social justice pedagogies included in this paper could be interpreted as being humanistic education where individual needs are addressed, while the structures that reproduce inequality are unchallenged [72]. As such, we acknowledge both the difficulty of enacting social justice pedagogies and that social justice pedagogies may not always transform structures nor make a uniform difference to all students [70]. However, we take strength by drawing on Michel Foucault’s [73] focus on the importance of small local resistances to unequal power relations as a way of bringing about social change. We are both encouraged and affirmed in our view that HPE teachers can make a difference when it comes to contributing to more equitable outcomes in the classroom and beyond.

## Figures and Tables

**Table 1:** Research participants and school context.

Teacher Name *:	Age:	Gender:	Years of Teaching:	Country:	SES **:	Students:
Tane	32	M	10	New Zealand	High	600
Dillon	32	M	7	New Zealand	Low	2200
Candice	32	F	9	New Zealand	Low	2200
Kendall	49	F	23	New Zealand	Low	650
Gary	42	M	19	New Zealand	Mid	1800
John	25	M	3	New Zealand	Mid	1800
Charlie	38	F	12	Sweden	Low	150
Emma	35	F	8	Sweden	Mid	200
Kane	30	M	6	Sweden	Low	350
Louise	45	F	20	Sweden	High	550
Ola	35	M	5	Norway	Mid	600
Kari	40	F	15	Norway	Low	100
Per	55	M	25	Norway	High	200

* All teacher names are pseudonyms; ** socioeconomic status; M: male; F: female.

**Table 2:** Observational template.

Observer	Date		
Description of school			
Description of teacher	Description of class		
* Captured incident	Description of teacher actions	Students’ actions	** Social justice issues
* Captured incident—an incident during the lesson that the observer believes is related to promoting social justice. A “captured incident” may be a moment through to a structure or theme that extends through the whole lesson.		** Prompts—these prompts may be useful in generating themes. They are designed to be used in column four on the observation template (when relevant).	
Critical issues of social justice ● Addressing gender issues ● Addressing issues of social class ● Addressing issues of ethnic and cultural equity ● Addressing issues of ability/disability ● Addressing issues of democracy ● Addressing issues related to understanding of the body ● Promoting social cohesion ● Other		Critical approaches to social justice ● Responsiveness to student needs ● Peer teaching ● Story telling of disadvantage or alternative perspectives ● Intentional use of non-dominant forms of HPE ● Problematizing traditional knowledge of the body ● Dialogical conversations—challenge dominant discourses ● Democratic classrooms ● Problem-posing ● Critical reflection (evident in interviews) ● Border-crossing/place-based pedagogies ● Naming issues of social justice ● Taking action against inequity	
